# Subunit Vaccines Using TLR Triagonist Combination Adjuvants Provide Protection Against *Coxiella burnetii* While Minimizing Reactogenic Responses

**DOI:** 10.3389/fimmu.2021.653092

**Published:** 2021-03-17

**Authors:** Alycia P. Fratzke, Sharon Jan, Jiin Felgner, Li Liang, Rie Nakajima, Algis Jasinskas, Saikat Manna, Fnu N. Nihesh, Sampa Maiti, Tyler J. Albin, Aaron P. Esser-Kahn, D. Huw Davies, James E. Samuel, Philip L. Felgner, Anthony E. Gregory

**Affiliations:** ^1^ Department of Microbial Pathogenesis and Immunology, Texas A&M Health Science Center, Bryan, TX, United States; ^2^ Vaccine Research and Development Center, Department of Physiology and Biophysics, University of California, Irvine, Irvine, CA, United States; ^3^ Pritzker School of Molecular Engineering, University of Chicago, Chicago, IL, United States; ^4^ Department of Chemistry, University of California, Irvine, Irvine, CA, United States

**Keywords:** *Coxiella burnetii*, guinea pig model, hypersensitivity, vaccine, TLR agonist, triagonist

## Abstract

Q fever is caused by the obligate intracellular bacterium, *Coxiella burnetii*, a designated potential agent of bioterrorism because of its route of transmission, resistance to disinfectants, and low infectious dose. The only vaccine licensed for human use is Q-VAX^®^ (Seqirus, licensed in Australia), a formalin-inactivated whole-cell vaccine, which produces severe local and systemic reactogenic responses in previously sensitized individuals. Accordingly, the U.S. Food and Drug Administration and other regulatory bodies around the world, have been reluctant to approve Q-VAX for widespread use. To obviate these adverse reactions, we prepared recombinant protein subunit vaccine candidates containing purified CBU1910, CBU0307, CBU0545, CBU0612, CBU0891, and CBU1398 proteins and TLR triagonist adjuvants. TLR triagonist adjuvants combine different TLR agonists to enhance immune responses to vaccine antigens. We tested both the protective efficacy and reactogenicity of our vaccine candidates in Hartley guinea pigs using intratracheal infection with live *C. burnetii*. While all of our candidates showed varying degrees of protection during challenge, local reactogenic responses were significantly reduced for one of our vaccine candidates when compared with a formalin-inactivated whole-cell vaccine. Our findings show that subunit vaccines combined with novel TLR triagonist adjuvants can generate protective immunity to *C. burnetii* infection while reducing reactogenic responses.

## Introduction

Q fever is a zoonotic disease caused by the obligate intracellular bacterium, *Coxiella burnetii*. Although overall mortality due to Q fever is low in humans, severe infections may cause atypical pneumonia, myocarditis, hepatitis, and spontaneous abortion (1–4). Symptomatic cases can also lead to chronic debilitating syndromes such as valvular endocarditis and Q fever fatigue syndrome in 1-2% of cases (1, 5, 6). *C. burnetii* is highly contagious by aerosol transmission, has a low infectious dose, and remains viable in the environment for long periods, making this agent a concern for bioterrorism (5). Q-VAX, a whole-cell, formalin-inactivated vaccine that provides long-term protection against *C. burnetii*, has not been approved for use in humans outside of Australia due to the high rate of local and systemic reactions in previously sensitized individuals. These reactions necessitate costly pre-vaccination serologic screening and intradermal skin testing which delays vaccination (7–9). Surveys for anti-*C. burnetii* antibodies in at-risk populations, particularly those who work in animal husbandry and veterinary fields, reported seroprevalence rates of 18.3-45.13% (10–12). The high seroprevalence rates show that these groups that would most benefit from vaccination also bear the greatest risk of hyper-reactive responses. Thus, there is a significant need to develop novel vaccine strategies against *C. burnetii* that provide long-term protection without inducing adverse reactions.

Subunit vaccines can provide a safer alternative to whole-cell vaccines with a lower risk of adverse reactions, but must overcome their lower immunogenicity with the use of adjuvants (13–15). The need for adjuvants to produce immunologic memory provides the opportunity to tailor immune responses to target antigens by altering adjuvant formulations. One class of adjuvant is toll-like receptor (TLR) agonists which activate innate immune receptors and induce proinflammatory cytokines through NF-κB transcription. Use of TLR agonists can mimic whole-cell vaccines which similarly activate TLRs through endogenous pathogen-associated molecular patterns (PAMPs) such as lipopolysaccharide (LPS), lipopeptides, and nucleic acids (13, 14, 16). By applying different combinations of TLR agonists to subunit vaccines, immune responses may be directed to enhance immunogenicity while minimizing adverse responses. Although individual TLR agonists have been associated with systemic toxicity secondary to diffusion from the vaccination site, conjugation of TLR agonists with polymers prevents rapid dispersal and markedly reduces this risk (17–19). Linking TLR agonists in diagonist and triagonist combinations has also been shown to alter and enhance immune responses by mimicking the spatial orientation of endogenous TLR agonists present on pathogens during infection (17, 18, 20).

In this study, we evaluated the protective efficacy and reactogenicity of five *C. burnetii* subunit vaccine formulations containing different linked (triagonist) or unlinked TLR agonist adjuvants in a guinea pig model of aerosol infection. Antigen and adjuvant combinations were selected based on the results of a previous publication (**Table 1**) (21). Our publication showed vaccines containing the triagonists TLR1/2_4_9a and TLR4_7_9a produced a greater Th1-biased immune response when compared to other triagonist adjuvants in both *in vivo* and *in vitro* experiments (18, 21). This Th1-biased response has been previously shown to be essential in vaccine-induced protection against *C. burnetii (* 22). AddaVax™, an MF59-like squalene oil-in-water emulsion adjuvant, was added to vaccine candidates because of its ability to enhance Th1-skewing (21). Similarly, a dendronized polymer (DP), was used as an adjuvant in one candidate vaccine because of its ability to enhance T cell responses to antigens (23).

**Table 1:** Formulations of vaccine candidates.

*Vaccine*	*Antigen*	*Adjuvant*
**A**	CBU1910, CBU0307, CBU0545, CBU0612, CBU0891, CBU1398	None
**B**	CBU1910, CBU0307, CBU0545, CBU0612, CBU0891, CBU1398	AddaVax™, DP, MPLA
**C**	CBU1910, CBU0307, CBU0545, CBU0612, CBU0891, CBU1398	AddaVax™, MPLA, CpG1018, 2Bxy
**D**	CBU1910, CBU0307, CBU0545, CBU0612, CBU0891, CBU1398	AddaVax™, Triagonist TLR4_7_9
**E**	CBU1910, CBU0307, CBU0545, CBU0612, CBU0891, CBU1398	AddaVax™, Triagonist TLR1/2_4_9
**F**	CBU1910, CBU0307, CBU0545, CBU0612, CBU0891, CBU1398	AddaVax™, pyrimido-indole, CpG1018, 2Bxy
**Sham**	PBS	None
**WCV**	Whole-cell Vaccine	None

Candidate vaccines contained 0.25 nmol of each antigen per vaccine dose. For adjuvants, AddaVax™ was dosed at 50% v/v, DP at 80 µg, and all TLR agonists at 2 nmol each. WCV was dosed at 25 µg of antigen per vaccine dose. Conjugated TLR triagonists were composed of Pam_3_CSK_4_ (TLR1/2), pyrimido-indole (TLR4), 2Bxy (TLR7), and CpG1018 (TLR9) as indicated.

In this report, we show that several vaccine candidates produce significant protection in a guinea pig model of aerosol infection with *C. burnetii* Nine Mile I (NMI). In addition, we identify a *C. burnetii* subunit vaccine using a TLR4_7_9 triagonist adjuvant that results in significantly less local reaction in pre-sensitized guinea pigs compared to a whole-cell vaccine (WCV) while providing similar protection from infection. Our results show how TLR triagonists may be utilized to modify immune responses to vaccination in favor of protective memory while limiting adverse hyper-reactive lesions.

## Materials and Methods

### *C. burnetii* Antigens

Six *C. burnetii* antigens, CBU1910, CBU0307, CBU0545, CBU0612, CBU0891, and CBU1398, were expressed in *Escherichia coli* BL21 cells, and purified by multiple column chromatography and endotoxin removal procedure at Genescript (Piscataway, NJ). Two antigens, CBU0612 and CBU1910 contained a His-tag. Purified proteins were evaluated by SDS-PAGE gel, Bicinchoninic acid (BCA) protein assay, and endotoxin removal confirmed using a Limulus Amebocyte Lysate (LAL) assay (MPL Laboratories, Sparta, NJ) (data not shown).

### TLR Triagonists and Adjuvants

The TLR triagonists were designed and generated as previously described (18). In brief, TLR triagonists were formed by bioconjugation reactions (amide bond formation, maleimide-thiol Michael addition, and azide-alkyne click chemistry) of individual TLR agonists to a central triazine core. The agonists used in this study include monophosphoryl lipid A (MPLA, TLR4), pyrimido-indole (TLR4), CpG ODN 1018 (TLR9), 2Bxy, an imidazoquinoline derivative (TLR7), and Pam_3_CSK_4_ (TLR1/2). Pyrimido-indole, CpG, 2Bxy, and Pam_3_CSK_4_ were used to form the linked triagonist adjuvants indicated in **Table 1**. TLR triagonist adjuvants, pyrimido-indole, 2Bxy, Pam_3_CSK_4_, and the imidazoquinoline derivative were purified by either high performance liquid chromatography or gel extraction and their masses were confirmed by MALDI-TOF or electrospray ionization-mass spectrometry (data not shown). CpG ODN 1018 (IDT, Coralville, IA) and MPLA (Avanti Polar Lipids, Alabaster, AL) were commercially purchased. Additional adjuvants used in our candidate vaccines include the squalene oil-in-water emulsion, AddaVax (Invivogen, San Diego, CA), and DP, a dendronized polymer analog (24, 25). Endotoxin in pyrimido-indole was quantitated using limulus amebocyte lysate (LAL) assay. All other lab-prepared adjuvants were analyzed for endotoxin contamination with Endotoxin Assay Kits (Genscript, Piscataway, NJ) or HEK TLR4 reporter cell assay (Invivogen, San Diego, CA). Antigen-adjuvant combinations were chosen based on previously published immunogenicity and challenge experiments (21).

### *Coxiella burnetii* Strains and Growth

For intratracheal infection, *C. burnetii* Nine Mile phase I (NMI) clone 7 (RSA493) was grown in embryonated yolk sacs and purified using gradient centrifugation as previously described (26). Experiments involving *C. burnetii* NMI were performed in biosafety level 3 (BSL3) facilities at Texas A&M Health Science Center. For formalin-inactivated whole-cell vaccine (WCV), live NMI cultures grown in ACCM-2 media as previously described were inactivated with 2% formalin for 48 hours (27).

### Guinea Pigs

Hartley guinea pigs, weighing 350-450 g, were purchased from Charles River Laboratories (Wilmington, MA). Guinea pigs were individually housed in microisolator cages under pathogen-free conditions and provided with free access to water, pelleted feed, and hay. Animals were housed in approved animal biosafety level 3 (ABSL-3) facilities and all experiments were performed under an animal use protocol approved by the Institutional Animal Care and Use Committee at Texas A&M University. Prior to infection, an IPTT-300 thermal transponder (Bio Medic Data Systems) was placed within the subcutis between the shoulder blades and temperatures were measured for 1-3 days to obtain individual baselines.

### Challenge Experiments

For challenge experiments, guinea pigs were administered candidate vaccines, WCV, or PBS (Sham) in 100 µL sterile PBS by intramuscular injection in the semitendinosus and semimembranosus muscles (**Table 1**). A boost vaccine was given in the opposite hindlimb two weeks later. Blood was collected from the lateral saphenous vein on days -3, 7, and 21, and 45 post-prime vaccination. Seven weeks after prime vaccination, guinea pigs were intratracheally infected with 10^5^ genomic equivalents (GE) of live *C. burnetii* as previously described (26). Briefly, animals were anesthetized with an intraperitoneal injection of 100 mg/kg ketamine and 10 mg/kg xylazine in PBS. A small animal laryngoscope (model LS-2; Penn-Century, Wyndmoor, PA, USA) was used to visualize the larynx then a MicroSprayer aerosolizer model IA-1B device was inserted to administer the bacteria in 50 µL of PBS. Guinea pigs were monitored daily for clinical signs including weight and temperature measurements. Five guinea pigs were utilized for each experimental group.

### Hypersensitivity Experiments

For hypersensitivity experiments, guinea pigs were infected with 10^6^ GEs of *C. burnetii* and monitored for two weeks post-infection as above then rested for an additional four weeks. To elicit hypersensitivity responses, two or four approximately 2-3 cm areas were shaved using electric clippers and depilatory cream on the right and left flanks. A vaccine candidate or WCV was then injected into the subcutis at each of the shaved sites (3 candidates and WCV per guinea pig). Temperature, weight, and reaction sites were monitored daily for two weeks. Each vaccine candidate was evaluated in four or five guinea pigs. Four uninfected guinea pigs were used as controls for unsensitized WCV and PBS responses.

### *C. burnetii* Array Composition

Purified His-tagged *C. burnetii* proteins were purchased (GenScript, Piscataway, NJ, USA) and purified lipopolysaccharide (LPS) from NMI was produced using the hot phenol method previously described (28). Purity of the LPS was confirmed using a SDS-PAGE gel that was subsequently silver stained (data not shown).

*C. burnetii* protein microarrays were produced as previously published with modifications (29). Briefly, purified proteins were mixed with array printing buffer and then printed onto nitrocellulose coated glass slides with an Omni Grid 100 microarray printer (Genomic Solutions). Serum samples were diluted to 1:100 and mixed with *E. coli* lysate (GenScript, Piscataway, NJ) and a His-tag-containing peptide (HHHHHHHHHHGGGG) (Biomatik, Wilmington, DE) to a concentration of 0.1 mg/ml, and pre-incubated at room temperature for 30 min to block any anti-His antibodies generated by the immunizations. Meanwhile, arrays were rehydrated for 30 min in blocking buffer. Arrays were probed with pre-incubated serum or PBS with 0.05% Tween (negative control) overnight at 4°C. Arrays were washed with TBS plus 0.05% Tween 20 then probed with anti-mouse IgG with streptavidin-conjugated Qdot^®^800 (Cat #Q10171MP, Thermofisher) at 1:200 dilution. Array images acquired using an ArrayCAM^®^ Imaging System (Grace Bio-Labs, Bend, OR, USA) to measure relative signal intensity corrected for negative control arrays.

### Quantification of *C. burnetii* Bacterial Loads

At two weeks post-infection, guinea pigs were euthanized and spleens and lungs were collected in for quantification of *C. burnetii* genomic DNA (gDNA) using qPCR (26). Tissues were homogenized and DNA extracted using the High Pure PCR template preparation kit (Roche, Basel, Switzerland) according to the manufacturer’s recommendations. Samples were analyzed on a StepOne Plus real time PCR System (Applied Biosystems) using com1-specific primers and probe (com1_L1 [CGCGTTGTCTTCAAAGAACT], com1_R1 [GCGTCGTGGAAAGCATAATA], and 5=-6-carboxyfluorescein-[FAM]-CGGCCAATCGCAATACGCTG-3’-6-carboxytetramethylrhodamine [TAMRA]).

### Histopathology

Lungs and skin sites from guinea pigs were fixed in 10% neutral buffered formalin for 72 hours at room temperature. For lungs from challenge experiments, four transverse sections were cut from the right lung (two from the caudal, one from the middle, and one from the cranial lobes). For vaccination sites, two to three sections were cut containing the epidermis to the underlying abdominal or intercostal muscle. Trimmed tissues were submitted to AML Laboratories (Jacksonville, FL, USA) for processing, embedding, and sectioning at 5 µm before staining with hematoxylin and eosin (HE). Histopathology slides were de-identified and evaluated by an ACVP boarded pathologist. Histopathologic scoring was performed on a 0-6 scale (**Figures 4A** and **6A**).

### Statistical Analysis

Statistical analyses were performed with GraphPad Prism v7.0 (GraphPad Software, La Jolla, CA, USA). Results were compared using one-way or two-way ANOVA with Dunnett’s correction for multiple comparisons. Differences were considered significant if p-value ≤ 0.05 (*), ≤ 0.01 (**), ≤ 0.001 (***), or ≤ 0.0001 (****).

## Results

### Serum IgG Responses to Vaccine Candidates

**Figure 1:**
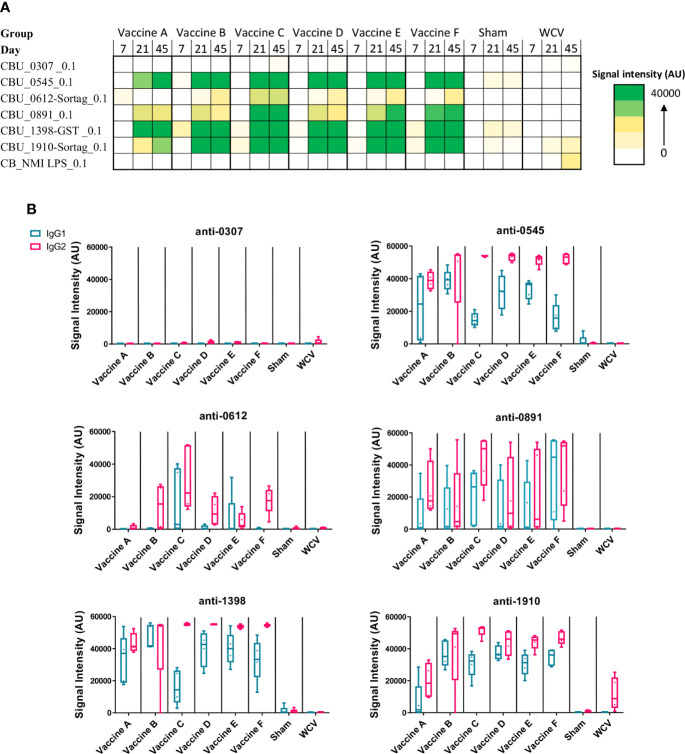
Antigen-specific serum IgG responses to vaccination. *C. burnetti* protein microarrays containing the six antigens from the vaccine candidates and NMI LPS. **(A)** Guinea pig sera collected from days 7, 21, and 45 post-prime vaccination were probed for total IgG. Values displayed as colorized scale are the means of the intensity from each group (n = 4-5 per group). AU, arbitrary units. **(B)** Day 45 samples were probed on the protein microarray for antigen-specific IgG1 (blue bar) and IgG2 (pink bar) subtype responses.

Specific IgG production to *C. burnetii* antigens has been shown to be an important mediator of vaccine-induced protection (30). The antigen combination in our candidate vaccines was chosen based on previous results evaluating serum from acutely and chronically infected humans which revealed seroreactive antigens as well as antigen-specific IgG responses to Q-VAX vaccination in mice (21, 31, 32). We measured antigen-specific IgG production in vaccinated guinea pigs to assess humoral responses to our vaccine candidates. Groups of five animals were given a prime vaccination on day 0 with a boost on day 14 and serum was collected from guinea pigs on days 7, 21, and 45 post-prime immunization. Samples were assessed by protein microarray for the six *C. burnetii* antigens in the vaccine formulations as well as purified phase I LPS (**Figure 1A**). Subsequently, day 45 serum samples were assessed for anti-specific IgG isotyping (**Figure 1B**).

All candidate vaccines, including the unadjuvanted vaccine A, induced robust IgG responses against CBU_0545, CBU_1398, and CBU_1910 by day 45. IgG production against CBU_0891 was more variable across vaccines, but all candidates induced significantly elevated serum IgG compared to Sham. Vaccine C produced the strongest response to CBU_0612, with milder responses in vaccines B, E, and F. None of the candidate vaccines resulted in significant IgG responses to CBU_0307, which is similar to our previously published data using mice (21). IgG isotyping for vaccines A to F were mostly IgG2-biased or IgG1/IgG2 balanced responses. The WCV vaccine group only induced significant IgG response to phase I LPS, with a mild response to CBU_1910 (mean signal intensity: 10937 ± 3042). WCV vaccination induced anti-CBU1910 IgG2-polarized response. As expected, none of the vaccine candidates produced anti-phase I LPS IgG.

### Subunit Vaccines Provide Protection Against Intratracheal *C. burnetii* Infection

**Figure 2:**
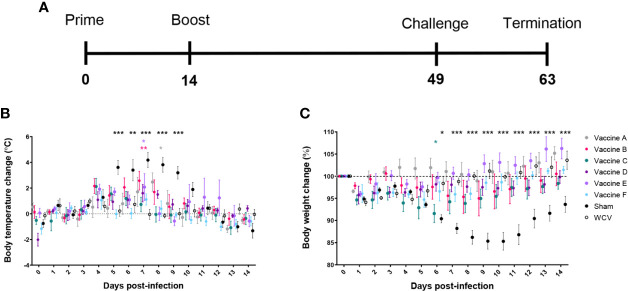
TLR agonist vaccines protect against weight loss and fever during aerosol infection. **(A)** Timeline of challenge experiment in days post-prime vaccination. Guinea pigs were challenged 7 weeks after prime vaccination. **(B)** Temperatures are presented as change from average individual temperature recorded prior to infection. **(C)** Change in starting body weight for 14 days post-infection. Body temperature and weight were monitored daily for 14 days post-infection. Graphs show the means for each group (n=5 per group) with error bars indicating the standard error of the mean. Data were analyzed using two-way ANOVA with Dunnett’s correction for multiple comparisons. Asterisks indicate significant differences between candidate- and WCV-vaccinated group (*p < 0.05, **p < 0.01, ***p < 0.001).

We assessed the protective efficacy of our candidate vaccines using a guinea pig model of aerosol infection (26). Guinea pigs were chosen for this study since they develop similar pulmonary disease compared to humans in response to aerosol infection and are more susceptible to acute infection than most immune-competent mouse strains (33–35). To evaluate protection, guinea pigs were vaccinated using the prime-boost schedule described above and rested for seven weeks post-prime prior to intratracheal infection with *C. burnetii* NMI (**Figure 2**). Five guinea pigs were assessed per experimental group. Guinea pigs were monitored daily for changes in weight and body temperature for 14 days post-challenge. WCV and sham vaccinated guinea pigs were used as positive and negative controls, respectively.

Sham-vaccinated guinea pigs had markedly elevated body temperature from days 4 to 10 after infection. All vaccine formulations provided significant protection against fever response compared to sham. Vaccines C, D, and F, which each contained TLR4, TLR7, and TLR9 agonists as a triagonist or unconjugated adjuvants, were able to mitigate any significant elevation in temperature compared to WCV (**Figure 2B**). All experimental groups showed some weight loss one to two days after challenge, which was presumed secondary to anesthesia required for intratracheal infection. Sham-vaccinated guinea pigs showed marked weight loss following infection which partially resolved by the end of the 14-day observation period. Similarly, guinea pigs vaccinated with vaccine C showed some transient but significantly greater weight loss compared to the WCV group at day 6 post-infection. Vaccine groups A, B, D, E, and F showed no significant decrease in weight compared to WCV-vaccinated guinea pigs. All vaccinated groups increased in weight by the end of the study period, except for vaccine C group which sustained a mild reduction in body weight (**Figure 2C**).

**Figure 3:**
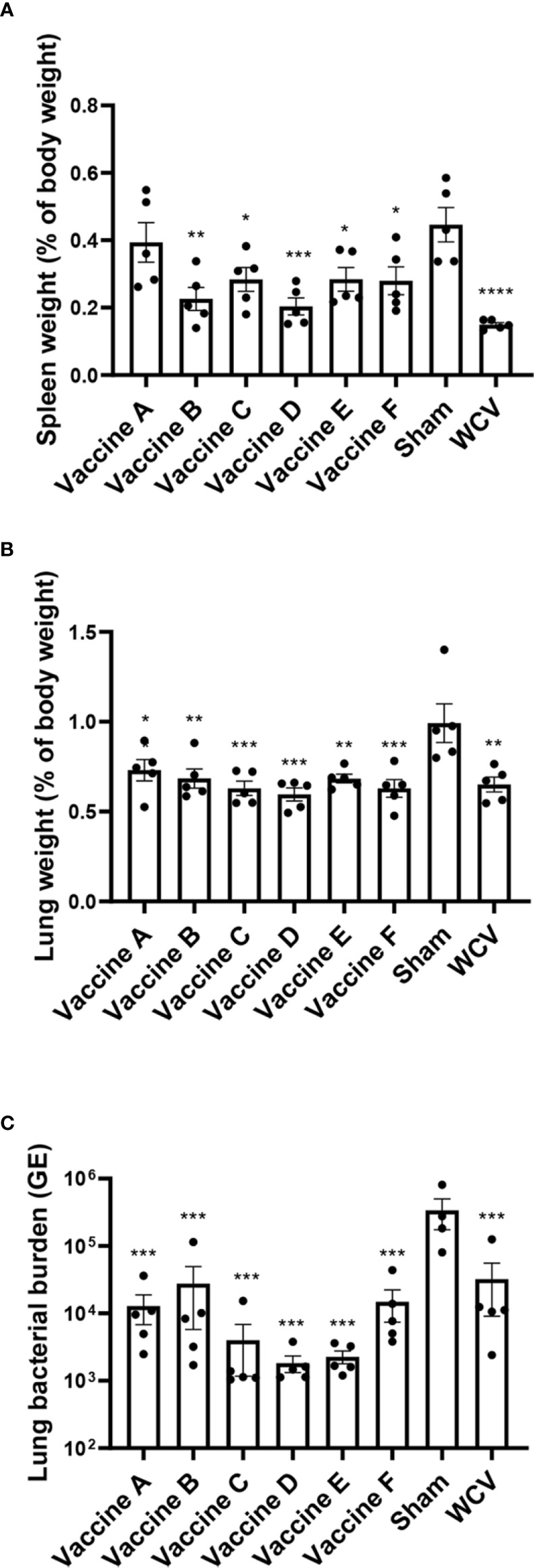
TLR agonist vaccination protects against splenomegaly and reduces pulmonary bacterial burden. **(A)** Mean spleen weight presented as a percentage of body weight. Vaccines B-F and WCV show significantly less splenomegaly compared to unvaccinated. **(B)** Mean lung weight presented as a percentage of body weight as an indicator of consolidation. Unvaccinated guinea pigs have significantly heavier lungs than vaccinated groups. **(C)** Mean genomic equivalents recovered from infected lungs show reduced bacterial burden in all vaccine groups compared to unvaccinated controls. Graphs show the means of each group (n = 4-5 per group) with error bars that represent the standard error of the mean. Data were analyzed using one-way ANOVA with Dunnett’s correction for multiple comparisons. Asterisks indicate significant differences compared to sham-vaccinated group (*p < 0.05, **p < 0.01, ***p < 0.001, ****p < 0.0001).

**Figure 4:**
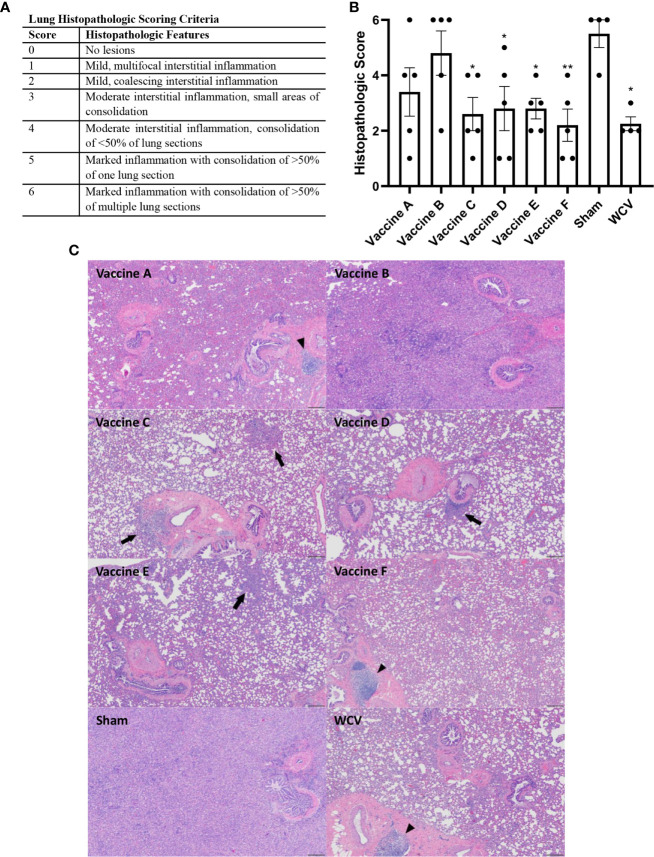
TLR agonist vaccines protect against pathologic lesions in the lungs. Histopathologic evaluation of the lungs 14 days after challenge. **(A)** Histopathologic scoring criteria for lung lesions. **(B)** Four representative sections of lung were examined and scored for severity of lesions. **(C)** Representative images of lungs from each experimental group showing foci of consolidation (black arrows) and BALT hyperplasia (arrowheads). Vaccine B and Sham lungs show consolidation of >80% of lung section. 4x magnification, HE stain, scale bar=200 µm. Graphs show the means of each group (n = 4-5 per group) with error bars that represent the standard error of the mean. Data were analyzed using one-way ANOVA with Dunnett’s correction for multiple comparisons. Asterisks indicate significant differences compared to sham-vaccinated group (*p < 0.05, **p < 0.01).

Animals were euthanized fourteen days after challenge, at which point spleens and lungs were weighed and collected for quantification of *C. burnetii* genome equivalents (GEs) and for histopathologic evaluation. All adjuvanted vaccine candidates and WCV provided significant protection against splenomegaly compared to sham-vaccinated guinea pigs (**Figure 3A**). Lungs were weighed as an indirect measure of pulmonary consolidation. Interestingly, all vaccine candidates had significantly lower lung weights compared to sham (**Figure 3B**). Bacterial burden in the lungs of all vaccinated groups was significantly less than sham, and comparable to WCV (**Figure 3C**). Pulmonary lesions were evaluated by histopathology using semi-quantitative scoring on a scale of 0-6 (**Figure 4A**). Sham-vaccinated guinea pigs produced severe granulomatous and lymphocytic inflammation frequently causing consolidation of >50% of lung sections. Vaccine groups C, D, E, F, and WCV showed significantly less pulmonary inflammation compared to sham-vaccinated controls (**Figure 4B**). Lung lesions in these groups consisted of mild to moderate interstitial infiltrates of macrophages, lymphocytes, and few heterophils with rare foci of consolidation. Guinea pigs immunized with vaccine B presented with severe consolidation of the lungs (>50% of lung section) often affecting multiple lung lobes, similar to sham-vaccinated controls. Guinea pigs in vaccine A group showed moderate multifocal consolidation with macrophages, lymphocytes, and heterophils (**Figure 4C**).

### TLR Triagonist Adjuvants Modulate the Reactogenicity of *C. burnetii* Vaccines

**Figure 5:**
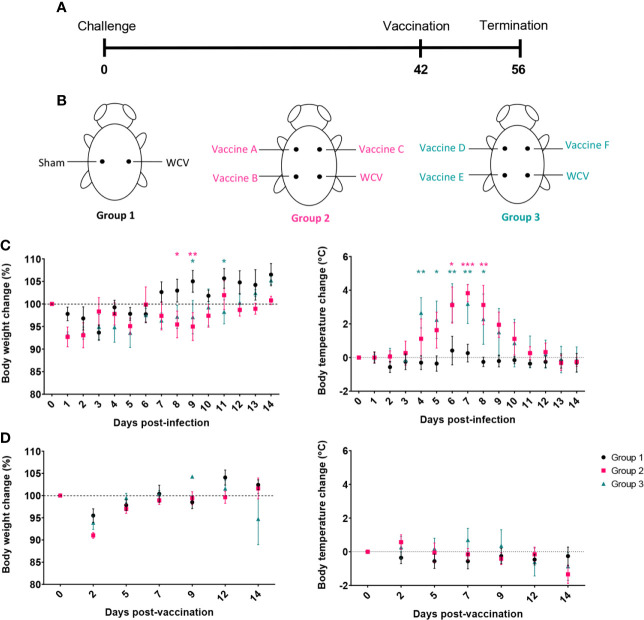
Guinea pigs were sensitized by aerosol infection prior to evaluation of the reactogenicity of TLR agonist vaccines. **(A)** Timeline of hypersensitivity experiment. Guinea pigs (Groups 2 and 3) were challenged by aerosol infection then rested for 6 weeks before SC injection with vaccine candidates. Uninfected guinea pigs (Group 1) were used to provide sham and unsensitized WCV skin sites as controls. **(B)** Location of injections for each experimental group. **(C)** Post-infection changes in body temperature and weight. Infected groups (2 and 3) show transient fever and weight loss compared to uninfected group (1). **(D)** Post-vaccination changes in weight and temperature. Graphs show the means of each group (n=4-5 per group) with error bars showing the standard error of the mean. Data were analyzed using two-way ANOVA with Dunnett’s correction for multiple comparisons. Asterisks indicate significant differences compared to uninfected group (Group 1) (*p < 0.05, **p < 0.01, ***p < 0.001).

Next, we evaluated reactogenic responses in guinea pigs with prior exposure to *C. burnetii* to subcutaneous vaccination with our candidate vaccines. Guinea pigs were intratracheally infected with 1x10^6^ GEs of *C. burnetii* NMI and then rested for six weeks before vaccination to mimic the most likely exposure route for pre-sensitization in humans (**Figure 5A**). Nine sensitized guinea pigs divided into two groups (Groups 2 and 3) were vaccinated with vaccines A-C and D-F, respectively, plus WCV (WCV+). Unsensitized guinea pigs (Group 1) were vaccinated with WCV (WCV-) and PBS (Sham) as controls (**Figure 5B**).

Body temperature and weight changes were monitored for two weeks post-infection and post-vaccination. Although all groups showed transient weight loss one to two days post-challenge due to anesthesia, infected guinea pigs displayed prolonged weight loss that resolved at 12-14 days post-infection. Infected guinea pigs also produced markedly elevated temperatures, up to 40.7°C, from days 4 to 10 post-infection indicating successful infection (**Figure 5C**). Uninfected control guinea pigs did not have any significant changes in body temperature during the 14-day observation period. None of the guinea pigs showed significant changes in body temperature post-vaccination. Similar to infection, guinea pigs lost weight during days 1 to 3 post-vaccination secondary to anesthesia, but maintained weight over the remainder of the observation period (**Figure 5D**).

**Figure 6:**
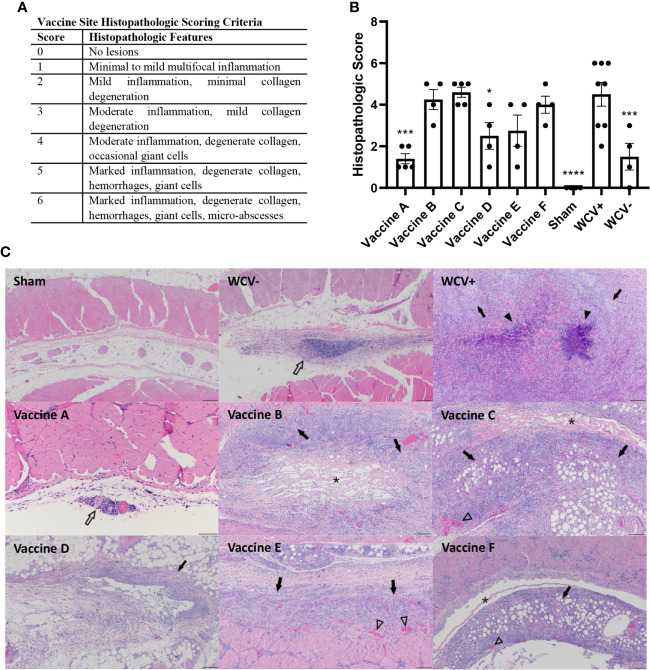
TLR agonist vaccines modulate local reactogenic responses to vaccination in pre-sensitized guinea pigs. **(A)** Histopathologic scoring criteria for vaccination site lesions. **(B)** Mean histopathologic scores for each experimental group. **(C)** Representative images of vaccine site lesions from each experimental group. WCV+ produces severe granulomatous inflammation (arrows) with multifocal micro-abscesses (arrowheads). Vaccines B, C, E, and F produce moderate to severe granulomatous inflammation (arrows) with areas of degenerate collagen (asterisks) and occasional foci of hemorrhage (open arrowheads). Vaccine D shows mild lymphohistiocytic inflammation (open arrow). Vaccine A shows minimal perivascular lymphohistiocytic inflammation (open arrow) 4x magnification, HE stain, scale bar = 200 µm. WCV+: WCV vaccination in sensitized guinea pigs. WCV-: WCV vaccination in unsensitized guinea pigs. Graphs show the means of each group (n = 8 for WCV+, n = 4-5 for all other groups) with error bars that represent the standard error of the mean. Data were analyzed using one-way ANOVA with Dunnett’s correction for multiple comparisons. Asterisks indicate significant differences compared to WCV+ vaccinated site in the same group (*p < 0.05, ***p < 0.001, ****p < 0.0001).

To assess the presence of local reactogenic responses to vaccine candidates, histopathology of the vaccine sites was performed 14 days post-vaccination. Two to three sections of each vaccination site were assessed by semi-quantitative scoring on a scale of 0-6 (**Figure 6A**). Although all adjuvanted vaccines induced local inflammatory lesions, vaccine D produced significantly less local inflammation compared to WCV+ (**Figure 6B**). Sensitized guinea pigs vaccinated with WCV showed severe granulomatous inflammation with multifocal areas of necrotic heterophils (micro-abscesses) and degeneration of adjacent collagen and myofibers similar to previously published reports (36, 37). Vaccine D caused mild to moderate histiocytic and lymphocytic inflammation with rare Langhans-type giant cells which occasionally extended to the subjacent muscle. Vaccines B, C, E, and F induced moderate to severe inflammation composed of macrophages, lymphocytes, and Langhans-type giant cells contain up to 30 nuclei with areas of degenerate collagen and hemorrhage. These lesions extended into the overlying deep dermis and underlying abdominal musculature. Unsensitized guinea pigs induced mild lymphohistiocytic inflammation within the subcutaneous tissue in response to WCV. Vaccine A produced only rare perivascular lymphocytic infiltrates (**Figure 6C**). Interestingly, although small numbers of heterophils were present in vaccine lesions in all of the candidate groups, none of the candidate vaccines produced abscesses seen in WCV lesions, even in the most severe reactions.

## Discussion

*C. burnetii* is a zoonotic, facultative intracellular bacterium that can induce a wide-range of acute and chronic syndromes in humans and animals. Although a whole-cell, formalin-inactivated vaccine against *C. burnetii*, Q-VAX, provides long-term protection, adverse systemic and local reactions in previously sensitized individuals prevent wide-spread licensure and use (5, 8). In this report, we tested the protective efficacy and reactogenicity of subunit vaccines utilizing TLR agonist combination adjuvants as a safer alternative to WCV. We previously evaluated the protective efficacy of several antigens and TLR triagonist combinations in a mouse model of aerosol infection showing that vaccines containing the TLR triagonists TLR1/2_4_9 and TLR4_7_9 produced Th1-skewed immune responses and provided protection against aerosol challenge (21). To further this, we used a guinea pig model of aerosol infection to assess both protection and reactogenicity of five vaccine candidates using TLR agonist and triagonist combinations.

Our data showed that all of our vaccine candidates provided measurable levels of protection against aerosol challenge. Surprisingly, even the antigen-only control appeared to provide some degree of protection against pulmonary infection based on pulmonary bacterial burden but did not prevent systemic responses, as evidenced by splenomegaly. Considering all measured indicators of protective efficacy, vaccine formulations D and F showed the greatest efficacy by preventing fever, weight loss, and splenomegaly, reducing lung bacterial burden, and minimizing pulmonary inflammation. These vaccines are very similar, containing agonists against TLR4, TLR7, and TLR9, but use a triagonist adjuvant and individual agonists, respectively. TLR4, TLR7, and TLR9 are all inducers of Th1-skewed immune response, which has previously been shown to be critical for immunity to *C. burnetii* infection (13, 14, 22). For example, T cell-deficient and IFNγ-deficient mice have increased susceptibility to *C. burnetii* infection, and humoral and cellular evaluation of Q-VAX vaccinated mice revealed a Th1-skewed immune response (22, 38). Thus, vaccines D and F likely induced robust Th1 memory resulting in greater protection during challenge. This is supported by the IgG isotyping showing mostly IgG2-baised responses to specific *C. burnetii* antigens in serum samples from vaccinated guinea pigs.

Although vaccine C provided similar protection across multiple parameters, this candidate failed to prevent significant weight loss during challenge compared to WCV. This was interesting considering that vaccines C and F only differ in their TLR4 agonist, containing MPLA and pyrimido-indole, respectively. Pyrimido-indoles are considered less potent stimulators of TLR4 compared to MPLA but are structurally better suited for conjugation with triagonist cores (18, 39). Additionally, pyrimido-indoles induce TLR4 signaling *via* MyD88 whereas MPLA mainly induces signaling *via* the TIR-domain-containing adaptor protein inducing interferon-β (TRIF) pathway (40, 41). Activation of MyD88 leads to the production of pro-inflammatory cytokines, while TRIF activation causes formation of type I interferons (42). The roles of MyD88- and TRIF-signaling in induction of immune memory are complex, but several studies indicate that MyD88 signaling is important for formation of memory T cells. In a MyD88 KO mouse model, dendritic cell maturation and trafficking to local lymph nodes occurs, but the production of pro-inflammatory cytokines from MyD88 activation has been shown to be necessary for the subsequent formation of memory T cells (43). Similarly, MPLA induces clonal expansion of T cells similar to LPS; however, MPLA also promotes contraction and terminal differentiation leading to fewer memory T cells (40, 44). MyD88-independent signaling also leads to a higher frequency of Th2, rather than Th1, cells in the absence of IFNγ secretion (43, 45). This differential signaling between MPLA and pyrimido-indole may explain why vaccine F provided greater protection from challenge than vaccine C.

In contrast, vaccine B produced the least protection against challenge based on lung pathology and lung bacterial burden. Notably, this vaccine candidate was the only one to lack a TLR9 agonist, suggesting that TLR9 agonism is important for vaccine-induced protection against *C. burnetii*. TLR9 is an intracellular toll-like receptor which detects bacterial and viral DNA by the presence of unmethylated CpG motifs. Whole-cell vaccines, such as Q-VAX, retain nucleic acids which may act as endogenous TLR9 ligands (13). Signaling *via* TLR9 is a potent stimulator of cellular immunity, including memory CD8 T cells which are important in bacterial clearance during infection (13, 46). We, and others, have previously shown that adjuvanting *C. burnetii* vaccine candidates with a TLR9 agonist significantly improves their efficacy (Gregory et al. unpublished data) (18). TLR9 signaling is important in generating resistance to other intracellular bacteria including *Mycobacterium tuberculosis*, *Brucella abortus*, and *Salmonella enterica* and may be an essential mediator of vaccine-induced protection against *C. burnetii (* 47–49).

We next evaluated the reactogenicity of our candidate vaccines using a sensitized guinea pig model. Vaccine D induced significantly less local inflammation compared to WCV. Despite containing the same TLR agonists, this TLR triagonist in vaccine D showed decreased local inflammation compared to individual TLR agonist vaccine counterparts, vaccines C and F. Triagonist conjugations modulate the activity of individual agonists by reducing individual potency, decreasing diffusion from the vaccination site, enhancing cellular co-activation, and mimicking the distribution of endogenous agonists on pathogens (17, 19, 20). Although experiments show TLR triagonists reduce systemic inflammation by decreasing diffusion from the vaccination site, their effect on local reactions is not as well studied (19, 20). Conceptually, TLR agonist conjugations increase local inflammation by retention of immunostimulatory adjuvants, but the effects of conjugated TLR agonists on immune responses can vary depending on the TLR agonist combinations used (18). A comparison of linked versus unlinked TLR4, TLR7, and TL9 agonists showed the linked TLR triagonist induced less IL12p70 systemically compared to unlinked TLR agonists in mice, but significantly greater TNFα, IL6, and IFN-β in *in vitro* stimulation of bone marrow-derived macrophages which suggested the triagonist would induce greater local inflammation (18). However, this does not correlate with the histopathology results in our experiments as the linked triagonist vaccine produced less reactogenicity than the unlinked counterpart. Further investigation is necessary to understand how TLR agonist combinations and conjugations alter the risk of local reactogenic responses.

Surprisingly, many of our candidate vaccines produced local lesions with similar severity to WCV despite containing only six *C. burnetii* antigens. It has been suggested that phase I lipopolysaccharide (LPS) is responsible for reactogenicity, but our candidate vaccines do not contain LPS yet some formulations still produced reactive lesions (9, 37). The lack of local inflammation in response to the unadjuvanted vaccine indicates that *C. burnetii* antigens alone do not induce local reactogenicity. Local inflammation may be the result of innate responses to adjuvants within the candidate vaccine. However, similar adjuvants and TLR agonists have been evaluated in vaccine trials against influenza, human immunodeficiency virus, and tuberculosis and did not result in the prolonged local inflammation shown in our experiments (50–52). It should be noted that these publications evaluated reactogenicity using unsensitized mice or in human trials where prior exposure to the target antigen is unlikely. Thus, their effects in pre-sensitized individuals are not well understood. It is likely that the combination of one or multiple *C. burnetii* antigens combined with certain TLR agonist adjuvants is producing local reactive lesions in pre-sensitized animals.

An interesting observation from the reactogenicity results was that local reaction sites from all candidate vaccines lacked micro-abscesses present in WCV-vaccinated guinea pigs. Although heterophils were present in smaller numbers in the lesions, there were no foci of necrotic heterophils. Mammalian heterophils differ from neutrophils in their cytochemical staining of cytoplasmic granules but are functionally analogous (53). Neutrophils participate in both innate and adaptive responses, however, since abscessation is not present in the vaccination sites of unsensitized guinea pigs, this is more likely a modification of the adaptive response (54). Neutrophil recruitment and activation is associated with Th17 cell cytokines in adaptive immunity and stimulation of Th17 has been associated with TLR2 stimulation (55). Only vaccine E contained an agonist that activates TLR1/2 which similarly did not show histologic evidence of abscessation. However, as discussed above TLR agonists show reduced activity upon conjugation with a triazine core and so decreased TLR2 agonism may explain the altered local inflammation in reactogenic responses caused by our candidate vaccines (18).

In these experiments, we evaluated the protective efficacy and reactogenicity of five subunit vaccine formulations using TLR agonist and triagonist combinations against *C. burnetii*. We showed that all of our vaccine candidates provided a measurable degree of protection, while those using a combination of TLR4, TLR7, and TLR9 appeared to produce the most significant protection against aerosol challenge. Evaluation of the reactogenic potential showed that all of subunit vaccines produced a measurable degree of reactogenicity in previously sensitized guinea pigs. However, candidate vaccine D, containing a TLR4_7_9 triagonist adjuvant, produced significantly less local inflammation compared to WCV. This result shows our *C. burnetii* subunit vaccine containing the TLR4_7_9 triagonist adjuvant can induce protection while mitigating local reactogenicity. Further experiments are needed to evaluate protection and reactogenicity of our subunit vaccines in non-human primates prior to progression of this vaccine towards human clinical trials. Overall, the TLR triagonist platform produces a favorable alternate vaccine strategy compared to Q-VAX for providing protection against *C. burnetii.*

## Data Availability Statement

The data that were generated and analyzed to support the findings of this study are available upon request from the corresponding author.

## Ethics Statement

The experimental procedures in this study were performed in an AAALAC-certified animal research facility at Texas A&M Health Science Center. All animal procedures were approved by the Institute of Animal Care and Use Committee at Texas A&M University (animal use protocol # 0429 D).

## Author Contributions

Experiments were designed by AG, JS, PF, DD, and AF. AF performed animal experiments, analyzed data, and wrote the manuscript. AG performed animal experiments and analyzed data. RN, AJ, and SJ printed and probed protein microarrays and SJ and DD analyzed the data. LL was responsible for correspondence with funding agencies. JF formulated the TLR agonist vaccines. SMan, FN, SMai, TA, and AE-K designed and synthesized adjuvants and TLR agonists. All authors were involved in manuscript revision and editing. All authors contributed to the article and approved the submitted version.

## Funding

This research was supported by DTRA contract HDTRA1-16-C-0009. The views expressed in this article are those of the authors and do not reflect the official policy or position of the U.S. Department of Defense or the U.S. Army.

## Conflict of Interest

PF, DD, JF, LL, SJ, RN, and AJ own shares in Nanommune Inc. Nanommune does not sell the arrays described in this paper, nor funded any part of the work described herein. Neither Nanommune or its shareholders are likely to benefit from the results described in this publication.

The remaining authors declare that the research was conducted in the absence of any commercial or financial relationships that could be construed as a potential conflict of interest.
